# Breast-conserving surgery with or without radiotherapy *vs* mastectomy for ductal carcinoma *in situ*: French Survey experience

**DOI:** 10.1038/sj.bjc.6604968

**Published:** 2009-03-10

**Authors:** B Cutuli, C Lemanski, A Fourquet, B de Lafontan, S Giard, A Meunier, R Pioud-Martigny, F Campana, H Marsiglia, S Lancrenon, E Mery, F Penault-Llorca, E Fondrinier, C Tunon de Lara

**Affiliations:** 1 Radiation Oncology Department, Polyclinique Courlancy, 38 rue Courlancy, Reims 51100, France; 2 Department of Radiation Oncology, Centre Val d'Aurelle, Montpellier Cedex 34094, France; 3 Department of Radiation Oncology, Institut Curie, 26 rue d'Ulm, Paris 75231, France; 4 Department of Radiation Oncology and Pathology, Centre Claudius Regaud, 20-24 rue du Pont St Pierre, Toulouse 31052, France; 5 Department of Breast Surgery, Centre Oscar Lambret, Rue F. Combemale, Lille 59020, France; 6 Department of Breast Surgery, Centre Léon Bérard, 28 avenue Laennec, Lyon 69373, France; 7 Department of Breast Surgery, Centre René Gauducheau, Bd J. Monod, Nantes 44085, France; 8 Department of Radiation Oncology, Institut Gustave Roussy, 39 rue C. Demoulins, Villejuif 94805, France; 9 Department of Statistics, Sylia-Stat, 10 Bd Marechal Joffre, Bourg-la-Reine 92340, France; 10 Department of Pathology, Centre Jean Perrin, 30 Place H. Dunant, Clermont-Ferrand 63011, France; 11 Department of Surgery, Centre Paul Papin, 2 rue Moll, Angers 49036, France; 12 Department of Breast Surgery, Institut Bergonie, 229 Cours de l'Argonne, Bordeaux 33076, France

**Keywords:** breast carcinoma, ductal carcinoma *in situ*, mastectomy, breast-conserving surgery, radiotherapy, tamoxifen

## Abstract

From March 2003 to April 2004, 77 physicians throughout France prospectively recruited 1289 ductal carcinoma *in situ* (DCIS) patients and collected data on diagnosis, patient and tumour characteristics, and treatments. Median age was 56 years (range, 30–84). Ductal carcinoma *in situ* was diagnosed by mammography in 87.6% of patients. Mastectomy, conservative surgery alone (CS) and CS with radiotherapy (CS+RT) were performed in 30.5, 7.8 and 61.7% of patients, respectively. Thus, 89% of patients treated by CS received adjuvant RT. Sentinel node biopsy (SNB) and axillary dissection (AD) were performed in 21.3 and 10.4% of patients, respectively. Hormone therapy was administered to 13.4% of the patients (80% tamoxifen). Median tumour size was 14.5 mm (6, 11 and 35 mm for CS, CS+RT and mastectomy, respectively, *P*<0.0001). Nuclear grade was high in 21% of patients, intermediate in 38.5% and low in 40.5%. Excision was considered complete in 92% (CS) and 88.3% (CS+RT) of patients. Oestrogen receptors were positive in 69.8% of assessed cases (31%). Treatment modalities varied widely according to region: mastectomy rate, 20–37%; adjuvant RT, 84–96%; hormone treatment, 6–34%. Our survey on current DCIS management in France has highlighted correlations between pathological features (tumour size, margin and grade) and treatment options, with several similar variations to those observed in recent UK and US studies.

In France, 49.236 new breast cancers were diagnosed in women in 2004 (Allemand *et al*, 2008). Between 2000 and 2004, the annual incidence rate increased by 2%. However, the precise proportion of ductal carcinoma *in situ* (DCIS) in France is unknown. Presently, DCIS accounts for approximately 15–20% of all breast cancers (Baxter *et al*, 2004; Leonard and Swain, 2004). The French screening programme was introduced in several counties (*départements*) in 1989 and was progressively extended all over France within the framework of the National Cancer Plan in 2000. The national coverage was achieved in 2005. Ductal carcinoma *in situ* rate among abnormal screen-detected mammographies reached 13.8%. Ductal carcinoma *in situ* is treated by mastectomy or conservative surgery (CS) with or without radiotherapy (RT) (Morrow *et al*, 2002; Ceilley *et al*, 2004; Mokbel and Cutuli, 2006). The best option is still a matter of debate, with wide differences in practice observed across European countries and the United States (Ceilley *et al*, 2004; Mokbel and Cutuli, 2006). Moreover, important regional differences were also reported.

Up-to-date data on DCIS are not available for France. We therefore performed a national prospective survey of the diagnosis, histopathological features and treatment of pure DCIS in France and compared our findings with those from other countries. The methodology of this survey was similar to a study on invasive breast cancer conducted in 2001–2002 (Cutuli *et al*, 2006).

## Materials and methods

Our observational, prospective cross-sectional study was performed from March 2003 to April 2004. A total of 77 physicians took part in the study and were recruited in surgery, oncology and radiation oncology departments throughout France. Most of them participated in the first national survey on infiltrating breast cancer in 2001–2002 (Cutuli *et al*, 2006). Those physicians are coordinators of a local breast unit, or general or gynaecological surgeons involved in breast cancer care. Finally, they are usually good representatives of all French regions (urban and rural) and types of sanitary structures. Indeed, patients were managed in cancer centres (49% of patients), private clinics (33%), or university or general hospitals (18%), reflecting quite well the daily current practice in our country. All patients seen in each hospital were prospectively included, but obviously with a more important recruitment in several specialized centres (7 with more than 50 cases each).

Eligibility criteria were female gender, ⩾18 years of age, with a diagnosis of pure DCIS. Patients with microinvasive lesions, previous or synchronous contralateral breast cancer or other cancers were excluded.

We collected data on demographics (age, family history of breast cancer, menopausal status, hormonal replacement therapy (HRT)), clinical presentation, mammography findings, biopsy procedures, specimen characteristics as given by the pathology report (tumour subtype, size, grade, excision quality and hormone receptor status) and treatment (types of surgery, number of operations, adjuvant therapy (RT with or without boost and hormone therapy) (see form in Supplementary Information). All data were checked (exhaustive pathological forms, radiation doses and other treatments) by the first author in collaboration with two pathologists of the scientific committee (FP-L, EM) and the statistical team, especially regarding some cases of ‘incoherencies’. The individual survey forms were made by each physician who sent them to the data management centre.

### Statistical methods

We used SAS software (version 8.2, SAS Institute Inc., Cary, NC, USA).

To compare two groups, we used the *χ*^2^ test for categorical variables, the *t*-test for continuous variables and the non-parametric Mann–Whitney test when the assumption of normality was questionable. To compare more than two groups, we used the *χ*^2^ test for categorical variables, one-way analysis of variance for continuous variables and the non-parametric Kruskal–Wallis test when the assumption of normality was questionable. Three treatment groups were compared, namely CS alone (99 patients: 7.8%), CS+RT (797 patients: 61.7%) and mastectomy (393 patients: 30.5%). We also performed regional comparisons dividing the country in five regions, namely Paris, northwest, northeast, southeast and southwest. Finally, a multivariate analysis was included, allowing predicting the therapeutic choices, such as mastectomy *vs* CS as well as sentinel node biopsy (SNB) *vs* axillary dissection (AD) according to age, tumour size (both quantitative variables), or grade and the presence of necrosis (both qualitative variables). A stepwise procedure was used to select variables for the multiple logistic regression analysis.

## Results

### Patient data

A total of 1289 patients were evaluable. Median age was 56 years (range, 30–84). Age distribution was as follows: 4% below 40 years, 25% 40–49, 36% 50–59, 22% 60–69 and 13% over 70 years. A family history of breast cancer was present in 30% of patients.

In total, 816 (63.3%) patients were menopausal (median age, 50 years); 52.3% of them had undergone HRT for a median duration of 7.7 years.

### Presentation at diagnosis

In 87.6% of patients, DCIS was diagnosed by mammographic abnormality. Only 12% of patients had clinical symptoms, such as palpable mass, Paget's disease, serosanguineous nipple discharge and/or retraction; such clinical symptoms were present in 9.5% of CS groups (CS and CS+RT) compared with those in 17.3% of the mastectomy group.

### Mammography findings

Of 1235 abnormal mammograms, 1227 could be evaluated: 1012 (82.5%) showed microcalcifications only, 62 (5.6%) opacity, architectural distortion or increased density and 146 (11.9%) combinations of these features. Of 1156 mammograms that could be evaluated according to the Breast Imaging Reporting and Data System from American College of Radiology (BI-RADS) classification, 107 (9.2%) were BI-RADS 3, 720 (62.3%) BI-RADS 4 and 329 (28.5%) BI-RADS 5.

### Preoperative biopsy

Preoperative needle biopsy was performed in 61.8% of patients. Vacuum-assisted needle biopsy was performed in 66.4% of these patients and stereotactic core-needle biopsy in 33.6%.

### Histopathology

The tumour was excised as a single specimen in 94.4% of patients, but one or more re-excisions proved necessary in 37% of patients. Tumour size was evaluable in 1062 (82.4%) tumours: 431 (40.6%) ⩽10 mm, 291 (27.4%) 11–20 mm and 340 (32%) >20 mm. Median tumour size was 14.5 mm: 6 mm for CS, 11 mm for CS+RT and 35 mm for mastectomy (*P*<0.0001). Nuclear grade was low in 21%, intermediate in 38.5% and high in 40.5% of tumours.

Excision was considered complete (usually margins ⩾1 mm) in 92% of CS patients and in 88.3% of CS+RT patients. The corresponding percentages were 3 and 5.8% for doubtful excision, 4 and 2.4% for incomplete excision, with 1 and 3.5% for non-evaluable excision. Tumour-free margins were measured in 66.7% of CS patients and 70.3% of CS+RT patients. The margin was <1 mm in 10.6% (CS) and 12.1% (CS+RT) of patients, 1–3 mm in 21% of patients (whether CS or CS+RT), 4–10 mm in 51.5% (CS) and 57.1% (CS+RT) of patients, and >1 cm in 16.7% (CS) and 10.3% (CS+RT) of patients. Thus, we do not have any specific analysis of multifocality and multicentricity, but we have the notion of ‘residual tumour’ on the specimen in case of multiple surgery, maximal tumour size and final margin status to predict the best surgical option (especially mastectomy). Indeed, the definitions of multifocality or multicentricity are often very subjective and confusing, especially in case of several little foci in the same area, but each one separated only by few millimetres.

Steroid hormone receptors were assayed in 31% of tumours. Oestrogen receptors (ER) were positive in 70% of patients and progesterone receptors in 59.5%. Hormonal replacement therapy had no influence on lesion size, architectural subtype and hormone receptor status, but was significantly associated with tumour grade. Indeed, low-grade tumours were found in 26.2% of women who had undergone HRT and in 18.3% who had not. Tumours were high grade in 34.2% (HRT) and 47.6% (no HRT) of women (*P*=0.0004).

### Treatment

Out of 1289 patients, 896 (69.5%) underwent CS and 393 (30.5%) mastectomy. The 69.5% undergoing CS could be further classified into 7.8% who underwent just CS and 61.7% who received adjuvant RT (89%). Generally, 70% of the patients underwent one operation, 27% two and 3% three. Residual DCIS foci were found in 59% of patients undergoing a second or third operation. Mastectomy was performed in 40% of patients as a first option but in 50% after one or two lumpectomies, and in 10% after quadrantectomy. Most patients who underwent multiple operations ended up by having a mastectomy. The number of breast surgical procedures was significantly reduced if a preoperative biopsy (stereotactic core-needle biopsy and more specially vacuum-assisted needle biopsy) was performed (Table 1). The rate of AD was also significantly reduced by the preoperative biopsy use, whereas the rate of SNB was increased (Table 2). Tumour size, nuclear grade, necrosis and age significantly influenced treatment modalities (Table 3). Mastectomy was used to treat 41% of high-grade tumours *vs* 15.3% of low-grade tumours. Generally, 93% of the patients with tumours ⩽10 mm were treated by CS (84.5% with RT) and 63.2% of patients with tumours ⩾20 mm underwent mastectomy. Mastectomy rates were 43% for comedocarcinoma and 28% for other lesions (*P*<0.0001). Conservative surgery was used to treat 1.5% of comedocarcinoma and 9.5% of other lesion subtypes (*P*<0.0001). The multiple logistic regression analysis confirmed that the factors significantly influencing the mastectomy choice were tumour size (OR: 1.088), young age (OR: 0.975) and high or intermediate grade (Table 4).

Of 896 women who were treated by CS, 797 (89%) received adjuvant RT. The median dose administered to the whole breast was 50 Gy. A 10 Gy boost (median dose) was given to 49% of patients (48% if excision was complete and 63.1% in case of incomplete or doubtful excision (*P*=0.018).

Tumour size, nuclear grade, lesion subtype (comedo *vs* others) and treatment strongly influenced axilla treatment (Table 5). Generally, 21.3% of patients underwent SNB and 10.4% AD. The multiple logistic regression analysis showed that sentinel biopsy procedure rates were higher in case of mastectomy, comedocarcinoma subtypes, large tumour size and high-grade lesions (Table 6). The AD use was significantly and only correlated to the mastectomy option (OR: 5.83; IC 95%: 3.68–9.24, *P*<0.001).

Adjuvant hormone therapy was administered to 172 (13.4%) patients; 138 received tamoxifen (80%), 7 a luteinizing hormone-releasing hormone (LH-RH) agonist and 25 an aromatase inhibitor.

### Regional variations in practice

There are some variations among regions in histopathological diagnosis (e.g., percentage of high-grade lesions, comedocarcinoma subtype and ER assessment) as well as in treatments (Table 7). The mastectomy rate ranged from 35 to 50% for high-grade DCIS and from 38.5 to 50% for comedocarcinoma. It varied from 20 to 37% according to the region. There were also wide regional variations in the delivery of adjuvant RT (81–96%) and in the administration of adjuvant hormone therapy (6–34%). Mastectomy rates also varied according to the type of institution (37.4, 32 and 19.4% in cancer centres, academic or general hospitals and private clinics, respectively, *P*<0.0001). The corresponding CS+RT rates were 87.3, 81.3 and 94.2%.

## Discussion

Our survey was conducted to obtain comprehensive and unselected up-to-date data on diagnosis, assessment and treatment of DCIS in France. The 77 physicians who included patients are breast cancer specialists working in different types of institutions (cancer centres, clinics and hospitals), reflecting the current daily practice in our country. However, we may have a small overrepresentation in our study of large centres.

Since 2000, regional breast cancer screening programmes in France have targeted women aged from 50 to 74 years. Ductal carcinoma *in situ* was diagnosed by mammography in 87.6% of our patients, but only in 61.2% of patients under the age of 40 years. This 87.6% rate of mammographically detected DCIS is slightly higher than the mean 81.5% rate of infraclinical lesions found in a former review of 909 patients treated for DCIS in Bordeaux) (Barreau *et al*, 2005). French (Cutuli *et al*, 2005a, 2005b) and European (Rutgers, 2001) guidelines also recommend preoperative diagnosis. Our study confirmed that preoperative biopsy significantly decreased the number of breast surgical procedures as well as AD and should be performed routinely (Table 1).

Our mastectomy rate is identical to that reported by two recent American and English studies (30.5%) (Katz *et al*, 2005; Dodwell *et al*, 2007). In the United States, the overall mastectomy rate decreased from 43% in 1992 to 37% in 1995 and 28% in 1999 (Ernster *et al*, 2000; Baxter *et al*, 2004; Mokbel and Cutuli, 2006). However, this rate was much higher (58%) in a large series from the Netherlands Cancer Institute (Meijnen *et al*, 2008). Tumour size, grade and necrosis are important factors in the decision to perform mastectomy. In our series, mastectomy was performed in 10% of patients with DCIS <10 mm but in 72% of patients with DCIS >20 mm, in 11% of patients with low-grade DCIS but in 54% with high-grade DCIS. On the other hand, several recent papers have emphasised the high risk of local recurrence (LR) in young women after CS, with or without RT in both retrospective studies and randomized trials (Fisher *et al*, 2001; Solin *et al*, 2001; Cutuli *et al*, 2002; Fourquet *et al*, 2002; Vicini and Recht, 2002; Bijker *et al*, 2006). These data probably explain our 50% mastectomy rates in women under the age of 40 years.

In theory, there is no axillary lymph node involvement in DCIS, although a recent review has reported 1.4% involvement in 1621 patients with pure DCIS (Morrow *et al*, 2002; Leonard and Swain, 2004). There may, however, be a risk of associated microinvasive foci, especially when the lesion is extensive (McMasters *et al*, 2002; Moran *et al*, 2005; Tunon de Lara *et al*, 2008). This may be a reason why, even though axilla management is pointless, 10.4% of our patients underwent AD and 21.3% underwent SNB. The potential risk of axillary lymph node involvement was probably overestimated by several physicians. The AD practice was highly influenced by tumour size and nuclear grade (Table 5), but especially by the breast surgery type; indeed, the AD rate was 5% after CS *vs* 22.6% after mastectomy (*P*<0.001). The global AD rate recorded in an earlier study by French Cancer Centres, including 1223 patients treated from 1985 to 1996 was 51.3% (Cutuli *et al*, 2005a, 2005b). Such a steep decrease (from 51.3 to 10.4% in France) has already been observed in the United States, where the global AD rate decreased from 34% in 1992 to 15% in 1999 (*P*<0.001), but nevertheless still remained high (30%) in 1999 in mastectomy patients (Baxter *et al*, 2004). In another US study (Smith *et al*, 2006), AD rate ranged from 18 to 28% across different sites. At present, AD is useless and should be replaced by SNB. In our study, SNB rate was 12.3% after CS *vs* 41.7% after mastectomy (*P*<0.001). Finally, a recent French multicentric study (Tunon de Lara *et al*, 2008) including 116 large DCIS found 4 positive sentinel nodes (3.4%). Thus, SNB could be proposed in patients treated by mastectomy.

For most authors, complete tumour excision is the key factor in reducing LR after CS (Silverstein *et al*, 1999; Kell and Morrow, 2005; MacDonald *et al*, 2005), but can be difficult to achieve. However, there is no clear consensus on the definition of ‘complete’ excision and adequate margins (Schwartz *et al*, 2000; Kell and Morrow, 2005). Tumour size and excision quality are factors often omitted in the reports of randomized controlled trials (Bijker *et al*, 2001) and retrospective studies (Solin *et al*, 2001). In our recent national practice guidelines (Cutuli *et al*, 2005a, 2005b), we have defined complete excision as a resection with at least 3 mm circumferential margins. Approximately 76% of our patients underwent complete tumour excision according to this criterion. However, some teams consider a 2 mm margin to be adequate if the patient subsequently receives RT (Kell and Morrow, 2005).

In patients with tumour excision who did not receive RT, the 10-year LR rates ranged from 19 to 27.8% (Schwartz, 2002). In four randomized controlled trials, the LR rates after CS without RT were 32, 28, 26 and 22%, with follow-ups ranging from 52 to 120 months (Fisher *et al*, 2001; Hougton *et al*, 2003; Bijker *et al*, 2006; Holmberg *et al*, 2008). In a French study, the 7-year LR rate was 32.4% in patients not receiving RT (Cutuli *et al*, 2002). In two latest Dutch studies (Schouten Van Der Velden *et al*, 2007; Meijnen *et al*, 2008), the 8- and 10-year LR after CS alone were 15.6 and 25%, respectively. Whole breast irradiation at 50 Gy after CS for DCIS halved *in situ* and invasive recurrence rates in earlier quoted trials (Fisher *et al*, 2001; Hougton *et al*, 2003; Bijker *et al*, 2006; Emdin *et al*, 2006; Holmberg *et al*, 2008) and several large retrospective studies (Solin *et al*, 2001; Cutuli *et al*, 2002; Fourquet *et al*, 2002; Schouten Van Der Velden *et al*, 2007; Meijnen *et al*, 2008). On the other hand, RT administration after wide excision (margins >1 cm) of small low-grade lesions is questioned (Cornfield *et al*, 2004; MacDonald *et al*, 2005; Solin *et al*, 2006; Buchholz *et al*, 2007, Silverstein *et al*, 2007). However, this result should be weighed up against the 12% rate observed at 5 years in a series of low-risk patients (Wong *et al*, 2006). On the other hand, the Van Nuys Prognostic Index (VNPI), combining tumour size, grade and margin width, was used to select ‘low-risk’ patients not requiring RT (Silverstein *et al*, 1996), but was widely criticised in two large American and English studies including 222 and 237 patients, respectively (Boland *et al*, 2003; MacAusland *et al*, 2007). A recent Italian study (Di Saverio *et al*, 2008) including 259 patients also used VNPI and concluded in the absence of statistically significant advantage in the CS+RT *vs* the CS group. However, the CS+RT group had several unfavourable factors widely increasing LR risk, such as lesion size >15 mm (67 *vs* 30%) and excision margins <1 mm (29 *vs* 4%). These features clearly explain the similarity of LR rates with an ‘apparent’ inefficiency of RT (7.5% for CS and 9.5% for CS+RT). Despite this, other English studies still use VNPI in current practice (Gilleard *et al*, 2008). Our survey showed that 89% of patients receive adjuvant RT after CS. A recent study on 1140 screen-detected DCIS in the United Kingdom reported a 57% ‘planned RT’ after CS. The RT use was influenced by tumour size (less or more than 15 mm), the presence of comedonecrosis and, more particularly, nuclear grade, but paradoxically not by margins (Dodwell *et al*, 2007). Such differences may show the high confidence of France (and of many centres in the United States and partly the United Kingdom) in randomised trials and large unselected retrospective studies on RT after CS for pure DCIS. Besides, there is clear evidence that boost increases local control in invasive breast cancer, especially in women under the age of 50 years (Bartelink *et al*, 2001). A boost was given to 49% of our RT patients compared with 50–75% of patients in other studies (Cutuli *et al*, 2002; Fourquet *et al*, 2002; Solin *et al*, 2005). A recent study (Omlin *et al*, 2006) on 375 young women (⩽45 years) treated for pure DCIS showed 28 and 14% LR rates with and without a 10 Gy boost. This 50% LR reduction rate is identical to that in invasive BC. However, there are several limitations in this study, that is long recruitment period, lack of specification on margin status and types of surgery.

The NSABP-24 trial (Fisher *et al*, 1999, 2001) found a significant reduction in ipsilateral and contralateral tumours on combining tamoxifen with CS+RT, which was however not confirmed by the results of a randomized trial from the United Kingdom, Australia and New Zealand (Hougton *et al*, 2003). Tamoxifen was effective in patients with ER-positive tumours only (37% of the patients in the NSABP trial) but not in patients with negative margins, absence of necrosis and in women over 50 years (Allred *et al*, 2002; Fisher *et al*, 2002). It has some serious side effects (endometrial cancer and thromboembolic events) and various ‘minor’ others. In a study of 94 patients with DCIS, 20 (21%) discontinued tamoxifen because of side effects or complications (mainly hot flushes, fatigue, weight gain and gynaecological or digestive symptoms) (Yen *et al*, 2004). The tumours of 31% of our patients were assayed for ER and progesterone receptors. The positivity rate was 70% for ER and 60% for progesterone receptors, in line with the 64 and 57% rates, respectively, recorded in a review of 1920 patients (Baxter *et al*, 2004).

There were important regional differences in histopathological features as well as treatment in our study population (Table 7). The mastectomy rate ranged from 20 to 37% and the use of RT after CS from 82 to 96%. Tamoxifen use varied widely from 6% in the northwest to 34% in the northeast of France. On the other hand, the size of DCIS ‘selected’ for CS alone ranged from 2.5 to 8 mm. It is also important to point out that wide histopathological variations (partly unexplained) can induce various therapeutic procedures.

Physician discretion and habitual practice rather than actual differences in DCIS probably explain these variations. Indeed, in the above-mentioned UK study (Dodwell *et al*, 2007), among 69 breast screening units included in the study, wide variations in the use of adjuvant RT were observed, ranging from 0% in six units to 100% in two (median: 57.7%). The authors explain that such variations in the use of RT reflect the differences in the perception of the risk–benefit relationship in this treatment, and also probably some difficulties to have access to RT facilities (Dodwell and Crellin, 2006). Wide variations have also been recorded in the United States (Ceilley *et al*, 2004; Katz *et al*, 2005), especially in the mastectomy rates (range: 26–45%) and the use of RT after CS (range: 39–74%), and have been confirmed in a recent analysis of the SEER data (Smith *et al*, 2006). In a retrospective study on 727 patients with DCIS enrolled in the Ontario Breast Screening Program from 1991 to 2000 (Rakovitch *et al*, 2007), there were also significant regional variations in RT use after BCS, ranging from 43 to 71% regardless of tumour pathological features, surgeon and hospital volume. On the other hand, some physicians are afraid by possible RT side effects. However, with modern techniques, the ‘simple’ whole breast irradiation (with or without boost, but without nodal irradiation) delivering 50 Gy in 25 fractions (or equivalent dose) resulted in <1% of complications, whereas in case of invasive LR, 15–20% of the patients finally died by metastatic evolution (Mokbel and Cutuli, 2006; Silverstein *et al*, 1998). Moreover, in the study reporting the SEER results (Warren *et al*, 2005), LR rates after CS and CS+RT were 15 and 10.7%, respectively, with a significant increase in breast cancer-related deaths when RT was omitted: 2.7 *vs* 0.8% (*P*=0.02).

In conclusion, randomised controlled trials can provide some answers, even if they are not exempt from criticism (Bijker *et al*, 2002). Several selection biases have been noted in DCIS trials such as inclusion of benign or microinvasive lesions and lack of compliance with study criteria (lesion size/surgical margins, quality of excision and treatment modalities) (Mokbel and Cutuli, 2006). However, there are now very consistent data confirming the importance of RT to minimise LR risk rates after CS for most DCIS, such as shown in a large study including 23 547 women from the California Cancer Registry (Innos and Horn-Ross, 2008). The future trials should identify more clearly selected patients (i.e., older than 60 years, with low-grade lesions under 10 mm and clear margin over 10 mm), in which RT can be safely omitted, and also those with aggressive DCIS requiring mastectomy.

## Figures and Tables

**Table 1:** Correlation between number of extra surgical procedures and pre-operative biopsy (percent patients)

**Number of extra surgical procedures**	**No biopsy**	**SCNB**	**VANB**
*CS and CS*+*RT*	*n*=257	*n*=175	*n*=334
1	72.4	83.4	92.3
2 or 3	27.6	16.6	7.7
*Mastectomy*	*n*=138	*n*=91	*n*=163
1	10.1	53.8	58.3
2 or 3	89.9	46.2	41.7

Abbreviations: CS=conservative surgery; RT=radiotherapy; SNCB=stereotactic core-needle biopsy; VANB=vacuum-assisted needle biopsy.

*P*<0.0001 for each comparison between treatments according to χ^2^-test.

**Table 2:** Influence of pre-operative surgery on axilla surgery (% of patients)

	**No biopsy**	**Previous biopsy**	***P*-value**
Sentinel node biopsy	15.4	25	<0.0001
Axillary dissection	14	8.2	0.0009

**Table 3:** Influence of tumour size, nuclear grade, necrosis and age on treatment modality (number and percent patients)

	**CS**		**CS+RT**		**Mastectomy**		**Total**	
	***N***	**%**	***N***	**%**	***N***	**%**	***N***	**%**
*Tumour size (pT, mm)* ^a^								
⩽10	62	14.4	339	78.6	30	7	431	40.6
11–20	14	4.8	223	76.6	54	18.6	291	27.4
>20	5	1.5	120	35.3	215	63.2	340	32
								
*Grade* ^b^								
Low	56	20.9	171	63.8	41	15.3	268	21
Intermediate	30	6.2	322	65.7	138	28.1	490	38.5
High	12	2.3	294	57.1	209	40.6	515	40.5
								
*Necrosis* ^c^								
None necrosis	68	13.4	334	65.9	105	20.7	507	42
No comedo necrosis	15	6.6	136	59.6	77	33.8	228	19
Comedo necrosis	9	1.9	271	57.2	194	40.9	474	39
								
*Age (years)*								
<50	26	26.2	203	25.5	141	35.9	370	28.7
50–60	41	41.4	284	35.6	136	34.6	461	35.8
61–70	14	14.1	198	24.8	73	18.6	285	22.1
>70	18	18.2	112	14.1	43	10.9	173	13.4

Abbreviations: CS=conservative surgery; RT=radiotherapy.

Evaluable cases: ^a^1062 (82.4%); ^b^1273 (98.7%); and ^c^1209 (93.8%).

**Table 4:** Mastectomy option influencing factors: multiple logistic regression analysis by stepwise procedure

**Variable**	**WALD value**	***χ*^2^ *P*-value**	**OR**	**IC 95%**
Tumour size (mm)	187	<0.0001	1.088	1.075–1.1
Age (years)	9.09	0.0026	0.975	0.96–0.99
Low grade	6.94	0.0084	0.497	0.29–0.83

Abbreviation: OR=odds ratio.

**Table 5:** SNB and AD practice according to age, treatment and tumour characteristics

	***N***	**SNB %**	**AD %**	***P*-value**
*Tumour size (cm)* ^(1)^				
<10	431	11.1	5.6	
10–20	291	18.2	10.3	<0.0001
>20	340	36.3	17.3	
				
*Nuclear grade* ^(2)^				
Low	268	11.2	5.2	
Intermediate	490	18.2	10.4	0.0025
High	515	29.7	13.2	
				
*Lesion subtype* ^(3)^				
Comedo	272	31.6^a^	11.4^b^	<0.0001^a^
Other	851	18.1	10.3	0.6^b^
				
*Treatment*				
CS	99	7.1	4	
CS+RT	797	12.9	5.1	<0.0001
Mastectomy	393	41.7	22.6	
				
*Age (years)*				
<50	370	26.8^c^	10.3^d^	
50–60	461	20.4	10.2	0.0074^c^
61–70	285	15.8	11.2	0.5^d^
>70	173	20.8	9.8	

Abbreviations: AD=axillary dissection; CS=conservative surgery; RT=radiotherapy; SNB=sentinel node biopsy.

Missing values: ^(1)^ 227; ^(2)^ 16; ^(3)^ 166.

a *P*-value between lesion subtype (comedo *versus* other) for SNB.

b *P*-value between lesion subtype (comedo *versus* other) for AD.

c *P*-value according to age (<50 *vs* >50years) for SNB.

d *P*-value according to age (<50 *vs* >50years) for AD.

**Table 6:** Sentinel node biopsy influencing factors: multiple logistic regression analysis by stepwise procedure

**Variable**	**WALD value**	***χ*^2^ *P*-value**	**OR**	**IC 95%**
Mastectomy	30.58	<0.001	3.127	2.088–4.68
Comedocarcinoma	6.92	0.0085	1.64	1.13–2.38
Tumour size (mm)	9.57	0.002	1.014	1.005–1.023
Low grade	7.58	0.0059	0.438	0.243–0.788

Abbreviation: OR=odds ratio.

**Table 7:** Regional variations in histopathological features and treatment modalities (% patients)

	**Paris**	**Northwest**	**Northeast**	**Southwest**	**Southeast**
% of high-grade lesions	33	43	44	49	40
% of comedocarcinoma subtype	10	20	26	43	27
% ER assessment	14	12	60	53	31
CS	3	3	8	13	4
Median size (mm)^a^	8	2.5	5	4	7
CS+RT	77	60	57	61	63
Mastectomy	20	37	35	26	33
Median size (mm)^b^	40	30	42	27	40
IR	39	64	37	66	64
Hormone therapy	13	6	34	9	12

Abbreviations: CS=conservative surgery; ES=oestrogen receptor; IR=immediate reconstruction; RT=radiotherapy.

a In patients treated by CS alone.

b In patients treated by mastectomy.

## Appendix

### DCIS french survey

Other participants: Achard JL, Clermont-Ferrand; Amalric F, Marseille; Antoine EC, Neuilly; Audrin O, Toulon; Avigdor S, Orleans; Bobin JY, Pierre-Benite; Body G, Tours; Bonnier P, Marseille; Bons-Rosset F, Nimes; Botton A, Pontoise; Bouteille C, St Etienne; Brandone JM, Marseille; Bremond A, Lyon; Brunaud-Charra C, Vandoeuvre-les-Nancy; Cailleux PE, Tours; Campana F, Paris; Caquot LM, Reims; Cellier P, Angers; Charvolin JY, Lille; Chevelle C, Toulouse; Chirat E, Meudon-la-Forêt; Cohen M, Aubagne; Cohen-Solal C, Saint-Cloud; Cretin J, Ales; Cuisenier J, Dijon; Cuvier C, Paris; Dauce JP, Rouen; Dohollou N, Bordeaux; Doudi-Gaci Z, Nantes; Fayolle M, Fontainebleau; Fourneret P, Grenoble; Fric D, Grenoble; Jaubert D, Bordeaux; Jonveaux E, Charleville; Kamioner D, Trappes; Ladonne JM, Rouen; Lavie M, Montpellier; Leduc B, Brives la Gaillarde; Lefranc JP, Paris; Levy C, Caen; Lucas B, Brest; Mandet J, Lille; Marie G, Cherbourg; Mathevet P, Lyon; Mermet J, Chambery; Methin A, Metz; Michaux JP, Cucq; Mongodin, Montelimar; Namer M, Mougins; Orfoeuvre H, Bourg-en-Bresse; Pariente F, Niort; Payan R, Grenoble; Quetin P, Strasbourg; Remuzon P, Dax; Resbeut M, Toulon; Rohart-De Cordoue S, Lille; Romieu G, Montpellier; Routiot T, Nancy; Rozec C, Thiais; Salmon R, Paris; Sautiere JL, Besançon; Serin D, Avignon; Touboul E, Paris; Travade A, Clermont-Ferrand; Vaini V, Aix-en-Provence; Vilcoq J, Paris
