# Supplementary material for: Breast-conserving surgery with or without radiotherapy vs mastectomy for ductal carcinoma in situ: French Survey experience
Source: Br J Cancer. 2009 Mar 10;100(7):1048–54. doi: 10.1038/sj.bjc.6604968 (PMC2670007; doi:10.1038/sj.bjc.6604968)
Supplement: Supplementary Information [file 6604968x1.doc]

# DCIS FRENCH SURVEY FORM (PRACCIS STUDY)

**Addendum 1**

# IDENTIFICATION

**CENTER |_|_|_|_|_|_|_|_|_|_|_|_|_|_|_|_|_|_|_|_|_|_|_|**

**Patient’s intitials |_|_|_| |_|_|_|**

**Birth Date |_|_| |_|_| |_|_|_|_|**

**File Center number |_|_|_|_|_|_|_|**

## MEDICAL HISTORY

**Breast cancer in family : (0) (1) |_|**

**Ménopause : (if yes : Age) (0) (1) (9) |_| / |_|_|**

**Hormonal replacement treatement : (0) (1) (9) |_|**

**Estrogens (1) Estrogèns + progestins (2) Duration (years) |_| / |_|_|**

**DISCOVERY MODALITIES**

**Clinical (0) (1) |_|**

**If (1) : Lump (1) Retraction (2) Sero-sanguineous discharge (4) Paget (8) |_|_|**

**Radiological (0) (1) |_|**

**Side : Right (1) Left (2) Bilatéral (3) |_|**

**Multifocality : No (0) Yes (1) |_|**

**RADIOLOGY**

**Mammograpy :**

**Use of magnification views No (0) Yes (1) |_|**

**Abnormalities : (if multiple : sum)**

**Round Mass (1) Stellar Mass (2) Architectural Distorsion (4) Asymmetric Density (8) Microcalcifications (16) |_|_|**

**- Number of microcalcifications foci |_|**

**- Size of largest focus |_|_| Bi-Rads classification : |_|**

**Biopsy (s) Not Performed (0) Performed (1) |_|**

**If (1) : Microbiopsy (14-18 G) Date : |_|_| |_|_| |_|_|_|_|**

**If (1) : Macrobiopsy (8-11 G) : Date |_|_| |_|_| |_|_|_|_|**

**FIRST BREAST SURGERY**

**Lumpectomy (1) Quadrantectomy (2) Mastectomy (4) |_|**

**Date |_|_| |_|_| |_|_|_|_|**

Specimen radiography No (0) Yes (1) |_|

**Needle Localization No (0) Yes (1) |_|**

**SECOND BREAST SURGERY**

**No (0) Yes (1) |_|**

**Wide lumpectomy (re-excision) (1) - Quadrantectomy (2) - Mastectomy (4) |_|**

**Date |_|_| |_|_| |_|_|_|_|**

**THIRD BREAST SURGERY No (0) Yes (1) |_|**

**Wide lumpectomy (re-excision) (1) - Quadrantectomy (2) - Mastectomy (4) |_|**

**Date |_|_| |_|_| |_|_|_|_|**

**Immédiate Reconstruction No (0) Yes (1) |_|**

**Type : Muscular flap (1) - Prothesis (2) - Both (4) |_|**

AXILLA SURGERY

Sentinel Node Biopsy No (0) Yes (1) |_|

**Date |_|_| |_|_| |_|_|_|_|**

**Axillary Dissection No (0) Yes (1) |_|**

Date |_|_| |_|_| |_|_|_|_|

###### PATHOLOGY

**Excision Quality**

**Specimen orientation No (0) Yes (1) |_|**

**Inking of margins No (0) Yes (1) |_|**

**Re-Excisions No (0) Yes (1) |_|**

**Re-excision without residual tumor (0) Re-excision with residual tumor (1) |_|**

**Maximum tumor size (mm) |_|_|_|**

**Highest nuclear grade :**

**Low (1) Medium (2) High (3) |_|**

**Necrosis : Absent (0) Non Comedo type (1) Comedo type (2) Not specified (9) |_|**

**Architectural Subtype: (if mixed form, please sum)**

**Cribriform (1) – Papillary (2) – Micropapillary (4) – Solid (8)**

**Clinging (16) – Comedocarcinoma (32) – Not specified (99) |_|_|**

**Final margin status :**

**(After re-excision or if no re-excision was performed)**

**Positive (1) Uncertain (2) Negative (free margins (4) Not specified (9) |_|**

**If negative : ≤ 1 mm (1) , 1-3 mm (2) , 3-5 mm (4) , 6-10 (8), ≥ 10 mm (16) , NP (9) |_|_|**

**VAN NUYS Index : 3-4 (1) 5-6-7 (2) 8-9 (4) |_|**

**Associated lesions :**

**Ductal Atypical Hyperplasia (0) (1) (9) |_|**

**Lobular Atypical Hyperplasia (0) (1) (9) |_|**

**Lobular Neoplasia / Lobular Carcinoma in situ (0) (1) (9) |_| Radial scar (0) (1) (9) |_| Number of sampled nodes : |_|_|**

**Hormone receptors dosage : Not performed (0) Performed (1) |_|**

**Estrogens (1) (0) (9) |_| Progestins (1) (0) (9) |_|**

**Her 2 overexpression Not Performed (0) Performed (1) |_|**

**RADIOTHERAPY**

**Not Performed (0) Performed (1) |_|**

**Start Date : |_|_| |_|_| |_|_|_|_|**

**Final Date: |_|_| |_|_| |_|_|_|_|**

**Dose par fraction (Gy) |_|_|**

Total breast dose : (0) (1) |_| |_|_|

**Boost : (0) (1) |_|**

**Boost dose : |_|_|**

**Boost type : Photons (1) - Electrons (2) - Brachytherapy (4) |_|**

**HORMONOTHERAPY**

**No (0) Yes (1) |_|**

**Type :**

**Surgical or radiotherapy castration (1) LH-RH Agonist (2)**

**Tamoxifen(4) Aromatase Inhibitors (4) |_|_|**
